# Carcinogen-induced DNA structural distortion differences in the *RAS* gene isoforms; the importance of local sequence

**DOI:** 10.1186/s13065-021-00777-8

**Published:** 2021-09-14

**Authors:** Georgina E. Menzies, Ian A. Prior, Andrea Brancale, Simon H. Reed, Paul D. Lewis

**Affiliations:** 1 School of Biosciences and Dementia Research Institute at Cardiff, Cardiff University, Cardiff, CF10 3NX UK; 2 Department of Cellular and Molecular Physiology, Institute of Translational Medicine, University of Liverpool, Liverpool, L69 3BX UK; 3 School of Pharmacy and Pharmaceutical Sciences, Cardiff University, Cardiff, CF10 3NB UK; 4 Division of Cancer and Genetics, School of Medicine, Cardiff University, Cardiff, CF14 4XN UK; 5 School of Management, Swansea University Bay Campus, Swansea, SA1 8EN UK

**Keywords:** Sequence context, Mutation rates, RAS genes, Molecular dynamics, DNA, Structural distortion, Benzo[a]pyrene diol epoxide

## Abstract

**Background:**

Local sequence context is known to have an impact on the mutational pattern seen in cancer. The *RAS* genes and a smoking carcinogen, Benzo[a]pyrene diol epoxide (BPDE), have been utilised to explore these context effects. BPDE is known to form an adduct at the guanines in a number of *RAS* gene sites, *KRAS* codons 12, 13 and 14, *NRAS* codon 12, and *HRAS* codons 12 and 14.

**Results:**

Molecular modelling techniques, along with multivariate analysis, have been utilised to determine the sequence influenced differences between BPDE-adducted *RAS* gene sequences as well as the local distortion caused by the adducts.

**Conclusions:**

We conclude that G:C > T:A mutations at *KRAS* codon 12 in the tumours of lung cancer patients (who smoke), proposed to be predominantly caused by BPDE, are due to the effect of the interaction methyl group at the C5 position of the thymine base in the KRAS sequence with the BPDE carcinogen investigated causing increased distortion. We further suggest methylated cytosine would have a similar effect, showing the importance of methylation in cancer development.

**Supplementary Information:**

The online version contains supplementary material available at 10.1186/s13065-021-00777-8.

## Background

Sequence context is known to play an important role in the rate at which Nucleic Excision Repair (NER) excises lesions [1–4]. The subtle changes in both nearest and further neighbouring bases has been studied [5], however, the interactions between DNA-carcinogens and these base contexts is largely unknown. There are at least sixty carcinogens present in tobacco smoke [6] many of which are thought to cause cancer by the induction of DNA damage. This damage is frequently caused by the covalent bonding of tobacco carcinogens to DNA bases [7]. The local DNA sequence context and methylation status of the sequence impacts upon the carcinogen binding frequency, the level of damage, the rate of repair and therefore subsequent mutation pattern, or signature [8]. Alexandrov *et al.*, have published an extensive collection of studies determining the mutational patterns in cancer including where they associated mutational signatures with tobacco smoking [8]. They determined a signature dominated by C > A (G > T) mutations, a signature they are able to replicate in vitro with benzo[a]pyrene (BaP) exposure (cosine similarity = 0.94). How sequence context can influence this signature is unknown and in order to further understand how the local sequence context plays a pivotal role in this mutation frequency. Others have also proposed that G > T mutations are caused by derivatives of BaP [9–11] including metabolite trans( +)anti-benzo(a)pyrene diol epoxide (BPDE) (Additional file 2: Fig. S1) [10]. BPDE is a highly mutagenic DNA adducting carcinogen and may be the main DNA adduct causing the mutations found in smoking related lung cancer [12]. BPDE binds to guanine at the N2 position and forms a DNA adduct with multiple alignments within the helix [13]. The (K)-7S,8R,9R,10S + anti-B(a)PDE enantiomer (10S) DNA adduct is positioned in the minor groove pointing towards the 5’ end of the helix, impacting on bases neighbouring the adducting guanine.

Previously we have studied sequences from the *TP53* gene using the methods detailed in this paper [47]. All sequences in our previous study were methylated as *TP53* has been shown to by hypermethylated in cancer, they were also all from the same gene, not isoforms with very similar sequences. Here we have looked at the Ras family of oncogenes make for an interesting set of similar DNA sequence contexts in which local sequence context effects on mutation rates can be studied. The *KRAS, NRAS* and *HRAS* genes have mutational spectra which differ in lung cancer for smoker’s vs non-smokers [14]. As well as having differing expression rates in certain cell and tissue types the small changes across protein coding regions in these genes may play an important role in the cancer rates.

The *RAS* oncogenes are hypomethylated in cancer, and in their normal state act as switches in pathways regulating proliferation and cell survival [15]. In cancer, *KRAS* is the most frequently mutated of the three proteins where mutations have been observed in 22% of all tumours analysed [14]. This compares to an 8% mutation frequency for *NRAS* and 3% for *HRAS* [14]. Base substitution hotspots in the *RAS* genes occur most commonly at three highly conserved positions throughout all proteins: codons 12, 13, and 61 [16]. The *RAS* genes contain only small differences in DNA sequence context, especially at the highly-mutated codons 12 and 13. Whilst differences in biological outputs and relative protein abundance have been proposed as explanations for the preponderance of KRAS mutations, a largely unexplored area is the contribution of rates of mutagen targeting and DNA repair.

All Ras proteins are expressed at a similar rate in lung tissue, so differences may be due to a sequence context rather than tissue expression one. Lung cancer shows a high association with G:C > T:A [8] base substitutions leading to a G12C amino acid change and in vitro studies have shown G:C > T:A transversions have been associated with bulky DNA adduct formation by tobacco smoke products [17]. This specific mutation is most common in current smokers with incidence progressively declining to zero in former and never smokers [18].

When considering all cancer types, the number of base substitutions at the guanine in the first position of *KRAS* codon 12 is just 23% of the total number of substitutions across all codons. For lung cancer however, this frequency increases to 51%. The patterns are similar for just G:C > T:A transversions at this first base which are 14% of the total number of mutations for all cancer types increasing to 45% for lung cancer. This suggests that the aetiology of mutations at the guanine in the first position of codon 12 in *KRAS* is likely different between lung cancer and other cancer types. Feng *et al*., showed that, in normal bronchial epithelial cells, the first guanine in codon 12 has a binding affinity for BPDE in all *RAS* isoforms but that the affinity is much higher in *KRAS* [19]. They also showed however, that the binding affinity of BPDE for mutable guanines in codons 12 and 13 is also strong in *KRAS* but non-existent or very weak in *NRAS* and *HRAS.* This suggests that subtle sequence context variation across the isoforms strongly affects the ability of BPDE to form adducts at key mutable guanines.

The formation of DNA adducts would contribute to but not dictate the G:C > T:A mutation spectrum within the *RAS* isoforms. The rate at which DNA repair occurs for bulky BPDE adducts is a major determining factor in influencing the spectral patterns and position of mutation hotspots at guanines. Feng et al., showed a repair rate of BPDE adducts at codon 14 in *KRAS* to be twice that of the same adduct at codon 12 [19]. Furthermore, small differences in the sequence context can have a large effect on DNA structural distortion caused by BPDE adducts which could affect DNA repair efficiency [20]. The patterns of differing DNA structural distortion between *RAS* isoforms according to sequence context may hold the key to understanding the underlying causes of susceptibility of codons with mutation hotspots to a slow DNA repair rate and higher mutation rate. Why this phenomenon occurs is largely unclear. Feng *et al.* concluded that K12 mutates at a higher rate due to a combination of both preferential binding and poor DNA repair [19].

We used molecular dynamics (MD) to study the potential contribution of local sequence context and structural distortion to mutation frequency when a BPDE adduct is present at three commonly mutated codons across the three *RAS* genes: codons 12, 13 and 14. Our primary aim was to assess BPDE adduct-induced structural distortions of the DNA helix within and around each adduct site *RAS* isoform codons 12, 13 and 14. This data could help determine whether bulky adduct-induced distortions differ according to local sequence context at key mutation sites. Furthermore, the structural differences could help hypothesise why certain RAS mutation hotspots occur in lung tumours. We also studied the effect altering the thymine in the *KRAS* sequence by converting TpG to a CpG and a mCpG. This allowed investigation of the similarity between methylated cytosine and thymine bases, when adjacent to a BPDE adducted guanine. The presence of a methyl-group, in either thymine or methylated-cytosine has been previously investigated in relation to its effect on the structure of DNA [21–23]. Our results predict that BPDE adducted *KRAS* codon 12 has a more severe DNA structural distortion than the other hotspots and non-hotspot sites studied as well as showing similarities between methylated-cytosine and thymine and a lessening of distortion when TpG was converted to CpG at the adduct site.

## Results

Using RAS as a model system, we were able to investigate the phenomena of local DNA sequence context and its impact upon DNA repair and rates of cancer. Further to this we were able to investigate the impact of the 3’ adjoining DNA base to the adducted guanine. By switching the thymine natively found in the *KRAS* sequence for a cytosine and a methylated cytosine we were able to study the similarities between methylated cytosine and thymine, both of which have a methyl group attached to the fifth carbon. Using a combination of MD simulations and MVA, we have investigated structural distortion caused by a bulky adduct at guanines within mutational hotspots and non-hotspot sequences in *KRAS, NRAS* and *HRAS*. The conformational stability of each sequence was calculated as all atom root mean square deviation (RMSD) values for the entire structure flexibility was investigated with root mean square fluctuation (RMSF) and the overall bending angle of the axis. The disruption of hydrogen bonding at the adducted base was then assessed. The overall structural change was measured using helical parameters including intra- and inter-base pair rotational and translational movements as well as base pair axis provided measurements on the type of structural distortion at adduct sites. Finally, multiple factor analysis (MFA) was used to reveal relationships and differences between *RAS* isoforms at sites with varying sequence context and mutational potential.

### Conformational stability and the flexibility differences between sequences

RMSD values were calculated to monitor the stability throughout the MD simulations (Additional file 2: Table 1). These values were calculated as an average, relative to the starting structure, providing an indication of the flexibility of each sequence over the simulation time. Simulations were stable between 0.3 ns and 100 ns of simulation and it was this time period used for further analysis. The control and adducted sequences for the first 300 ps, had RMSD values of between 0.17 nm ± 0.02 and 0.21 nm ± 0.06 respectively. For the remainder of the simulation time the sequences had RMSD values in the range of 0.22 nm ± 0.01 and 0.29 nm ± 0.05. All differences in RMSD distributions between the adducted and control sequences were significant (P < 0.001), with values for adducted sequences being greater than control. This was to be expected, as the inclusion of a large bulky adduct should increase the movement within the DNA sequence. RMSD results show a general increase in the adducted sequences compared to the non-adducted controls. This would be expected due to the large BPDE adduct interfering with the helical structure and causing more fluctuation and movement. All differences in the distributions of RMSD values between adducted and control sequences were significant (P < 0.001). Further to this, we also calculated RMSF for each base throughout the sequences (Additional file 2: Fig. 3) a significant difference was found between K12,13 m and 14 controls and adducted sequences (P < 0.001), but no significant difference was found between the remaining sequences.

### Hydrogen bond quality index

The difference between ideal Watson–Crick hydrogen bonds and the values observed in our simulations were calculated using the hydrogen bond quality index (I_H_) for all adducted and control sequences [24]. Values for I_H_ show a deviation from ideal Watson–Crick bonding, with a range across both controls and adducted sequences of between 4.52 and 4.63. There were no significant differences between the hydrogen bond indexes of the sixth bases calculated for the control and adducted DNA sequences. The only increase in I_H_ between control and adducted sequence occurred in *KRAS* codon 12 (K12) all other sequences show a more ideal Watson–Crick binding pair for the adducted sequence, with the greatest decrease I_H_ observed in *KRAS* codon 14 (K14).

### Adduct-induced DNA structural distortion variability in the RAS gene sequences

For each of the *RAS* hotspot and non-hotspot sequences, helical parameters were calculated for 4851-time frames and the median for each parameter collected to provide insight into the sequence structural changes over time. Time frames spanned from 300 ps to 100 ns of real time. Median values, along with standard deviations, were visualised graphically so that sequences with structural differences could be identified. As with previous studies, the first and last base of each sequence were excluded from multivariate analysis due to the large fluctuations in structure. This left 9 or 8 data points for intra and inter helical parameters, respectively, and these were collated (for each of the 17 parameter types) and input into MFA. The first stage of MFA analysis includes a normalisation procedure to permit values from each independent sequence to be compared against all others. This also eliminates the possibility that angle measurements will dominate over distance.

**Fig. 1:**
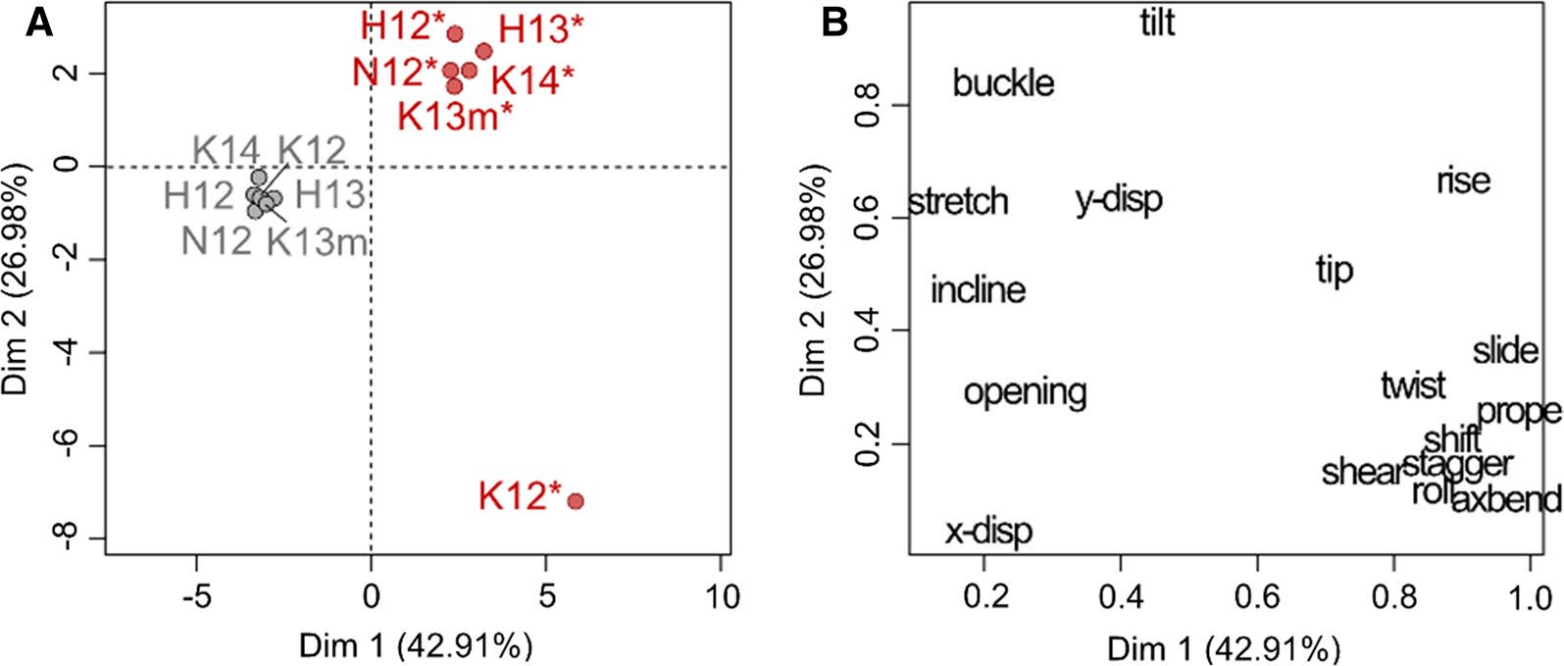
Multiple factor analysis of adducted and control DNA sequences. **A** The scatterplot shows how all adducted and control sequences correlate with the first dimension (Dim1) and the second dimension (Dim2). Sequence labels end in ‘*’ to denote the 10S BPDE adduct. **B** The scatterplot shows how each of the structural parameters correlate with Dim1 and Dim2

The first two dimensions (Dim1 and Dim2) explained 42.91 and 26.98% respectively, of the variation contained within the structural parameter data set. The MFA provides a map of the similarities and variation in distortion observed within the sequences as well as an insight into which parameters cause the variation observed. A scatterplot of how each sequence correlates with Dim1 and Dim2, given the relative distortion, is shown in Fig. 1A. All control sequences vary little in structural movement over time and thus expectedly group closely together on the MFA map. Dim1 separates all control from adducted sequences where adducted sequences have a positive correlation and controls a negative correlation with this dimension. Dim2 further separates the sequences with adducted sequence K12 being the only adducted sequence to have a negative correlation with this dimension. All other adducted sequences have a positive correlation with Dim2 and all controls a negative correlation. Aside from the adducted K12 sequence, all other adducted sequences show very little variation in distortion (Additional file 1).

The contribution of each parameter to this apparent difference in distortion observed for K12 was determined using the correlation of each parameter on Dim1 and Dim2 (Fig. 1B). Dim1 separates the parameters into roughly two groups which likely have the greatest influence on structural distortion differences between adducted and control sequences. Given the pattern of separation seen for the sequences in MFA, the parameters having the greatest correlation with Dim2 were assumed to contribute to the distortion differences between adducted sequences. The intra-base parameter buckle and inter-base parameter tilt had a high correlation with PC2 and were investigated further to elucidate the differing post-adduction structural distortion observed for *KRAS* codon 12.

### Characterisation of DNA BPDE-induced distortion of KRAS codon 12

**Fig. 2:**
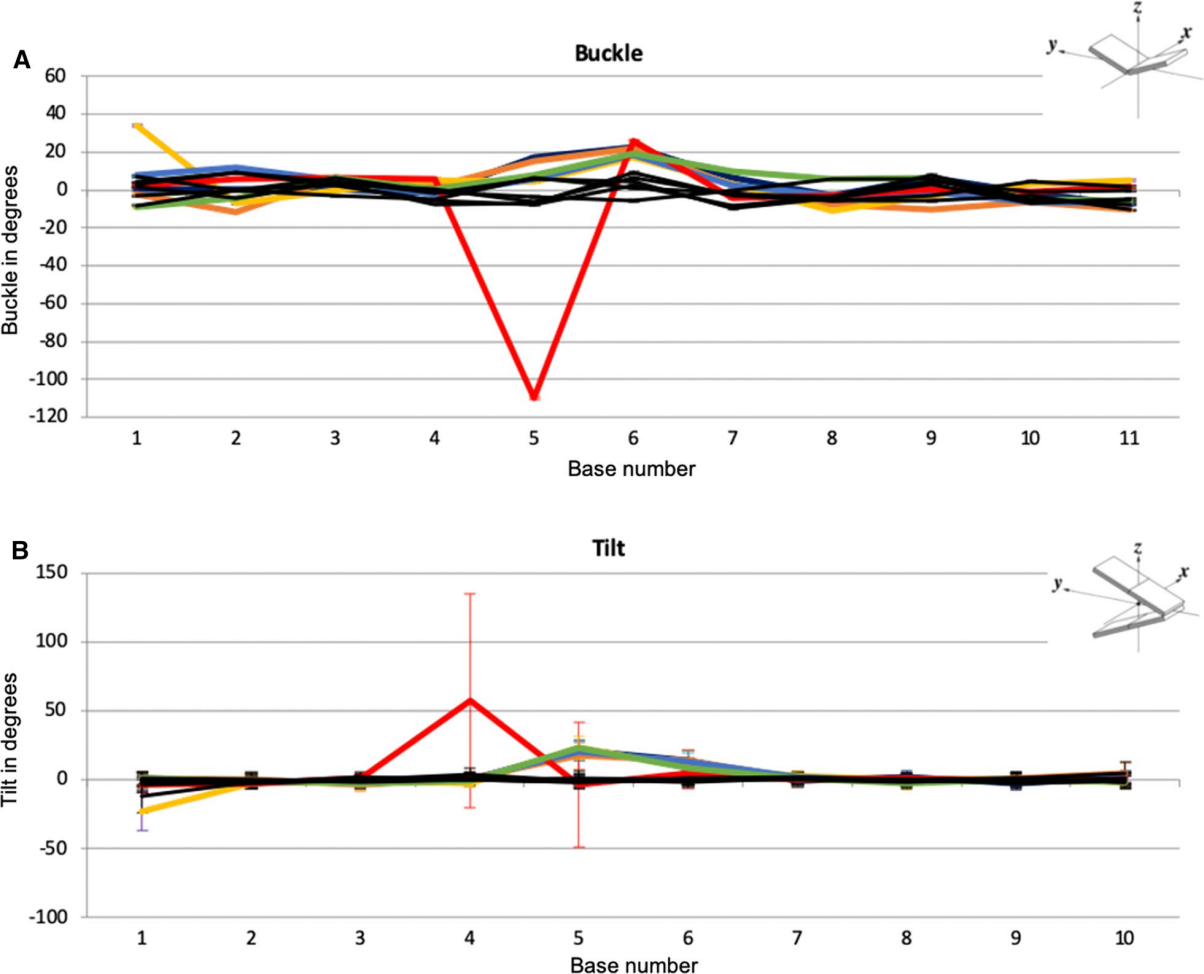
Line graph representations of average magnitude of rotation values for every base across each adducted and non-adducted control sequence. **A** Average buckle angle. **B** Average tilt angle. Parameter schematics re-printed, with permission, from [25]. Control sequences are black, **K12-red**, K14-orange, K13-purple, H12-indigo, H13-yellow, N12-green

The median values for the buckle and tilt parameters are depicted in line graphs in Fig. 2. The magnitude of rotation is displayed at each base for buckle, or between bases for tilt. The data represents the rotation of the base pair about an axis which is the short axis of the base pair and base pair step, respectively. The buckle rotation is increased in the graph indicating buckling towards the 3’ end for all adducted sequences at the fifth base position with the exception of K12, where the buckle is increased towards the 5’ direction. For all adducted sequences, except K12, there is an increase of tilt in the positive direction, towards the 3’ direction, for both the fifth base pair step and the sixth. K12, on the other hand, shows an increase in tilt at the fourth base pair step, and no increase in tilt at the fifth base pair step.

**Fig. 3:**
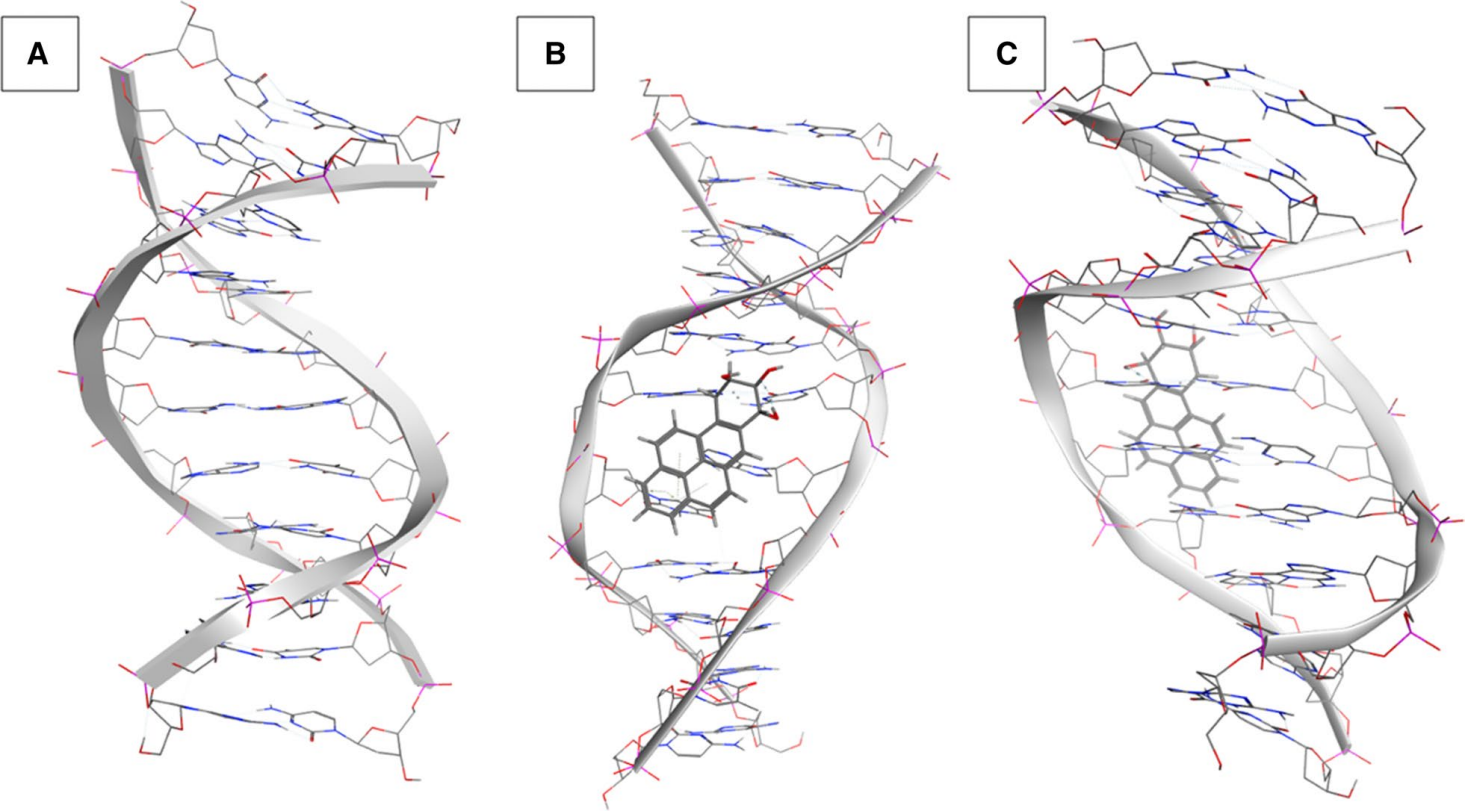
K12 control sequence (**A**) K12-adducted sequence (**B**) and K14-adducted sequence (**C**), images were taken from structures at the end of the MD simulations. (remaining adducted sequences can be seen in Additional file 2: Fig. S2)

We compared the distributions of the magnitudes of rotation for buckle and tilt over time, i.e. values for all time points for each sequence from 300 ps to 100 ns. Interestingly, the adduction rate for *KRAS* codon 14 (K14) has been shown to be higher than codon 12 (K12)[9] but has a much lower mutation rate in cancer. The level of distortion for K14 is similar to other sequences except K12 post adduction. If we look at the variation for the magnitude of rotation for the buckle parameter, there is also clearly a difference between the two codons when an adduct is present (Fig. 3A, B). The distribution in buckle values for K12 has little overlap between the control (median = − 7.96) and adducted sequences (median = − 109.14) whereas the K14 sequence differs little with median values of -4.06 and 6.25 for the control and adducted sequences, respectively. Thus, a much greater distortion is seen in the K12 codon relative to K14 and the degree of buckle remains relatively constant in both sequences. The tilt parameter for K12 shows a median value of -0.41 for the control, and -22.94 for the adducted sequence. We can see from Fig. 3C however, that the adducted sequence has a much greater variation in tilt values suggesting an increase in this type of movement in K12 relative to K14 (Additional file 2: Fig. 1) when the adduct is present. Codon K14 for the same parameter shows less overlap between the control (median = 1.38) and adducted (median = 23.59), with much less variability in the adducted sequence. The structural distortion surrounding *KRAS* codon 12 at the end of the MD simulation is represented in Fig. 3 where it is shown with the K12 control and the K14 adducted sequences for comparison.

### The contribution of the adjacent 3’ base to structural distortion at KRAS codon 12

**Fig. 4:**
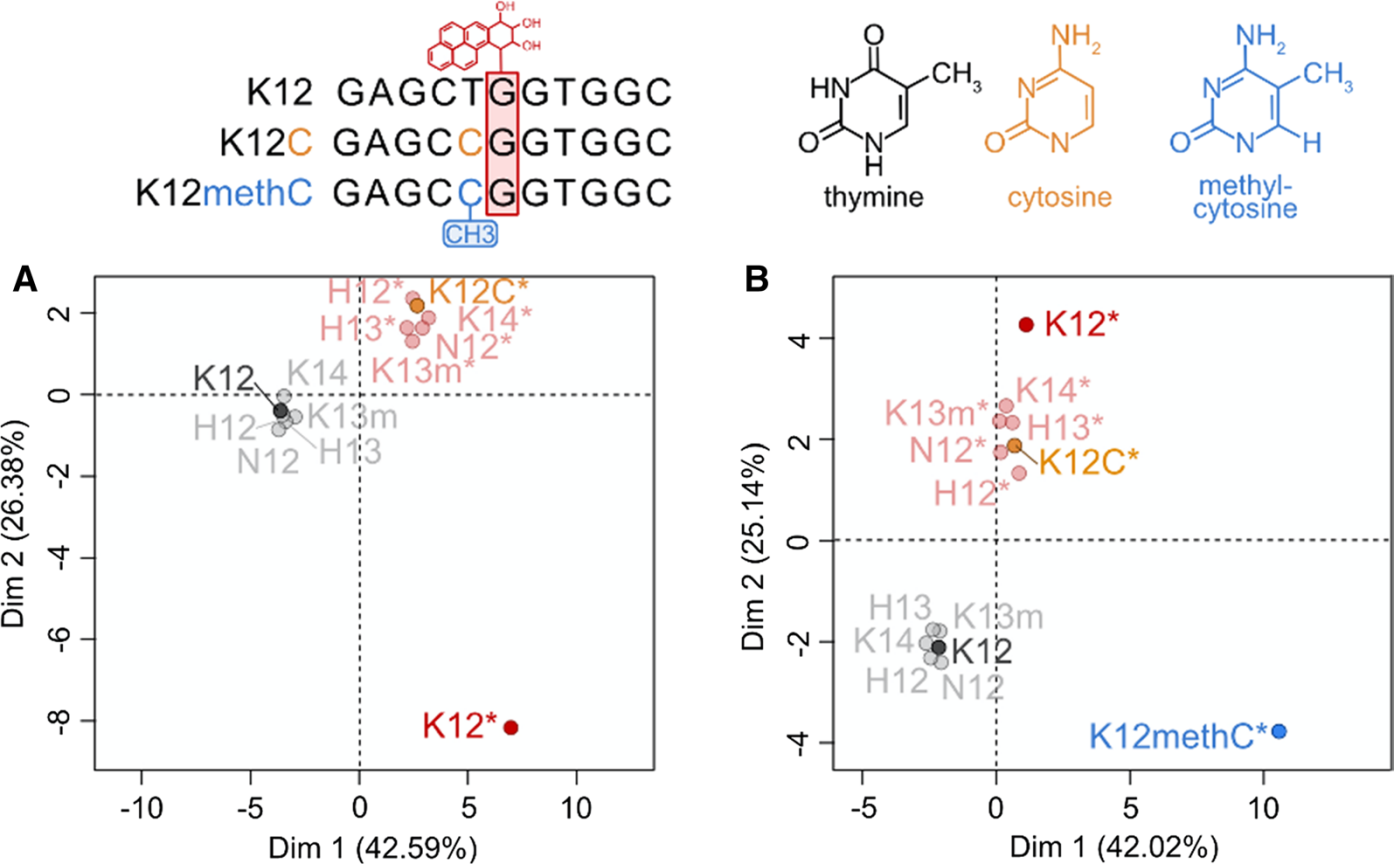
The scatterplot shows how all adducted and control sequences correlate with the first dimension (Dim1) and the second dimension (Dim2). Those is grey are controls and those in colour with a * are adducted sequences. In **A** and **B** K12C donates the altered K12 sequence containing a C in place of a T and in **B** K12MethC denotes the altered sequence containing a Methylated C in place of a T. The sequences of K12 are displayed above the charts with diagrammatic indicators of adducted sites

Given the extent of difference in post-adduct structural distortion occurring at K12 relative to other codons across isoforms it was clear that subtle variations in sequence context were a significant contributor, a figure showing the BPDE adduction site and distorted T5 base can be seen in Additional file 2: Fig. S4. To investigate further, we assessed the potential contribution of the base 5’ to the adducted guanine in K12. In the first instance, the thymine which precedes the adducted guanine 5’ in the K12 codon was substituted for a cytosine (K12C) providing a sequence identical to that of H12 (spanning from 3 bases away 5’ to the adducted guanine to the adjacent 3’ base (GCC**G**G)), we ran this for both a control and adducted sequence. Following further analysis by MFA, the resulting substitution led to a degree of distortion similar to that observed for other adducted sequences but different to the original K12 (Fig. 4A). Secondly, the MD simulation was repeated following a methyl group added to the substituted 5’ C in the K12C sequence (K12MethC), again control and adducted sequences were investigated. Following MFA, a different and more diverged pattern of structural distortion variation was revealed (Fig. 4B). Dim1 separated the adducted from control sequences as before but the K12MethC distortion led to the sequence having a strong negative correlation on Dim2 but the K12S sequence now had a positive correlation on Dim2. This suggested that the degree of distortion was much greater when a methyl group was present. Following analysis of the structural parameter data on the first two dimensions (results not shown), the buckle parameter was examined in more detail. The median buckle angle at the sixth base for sequences, K14, H12, H13 and N12 was 10.1° whereas buckle at this position for K12 was − 109.7°. The median buckle for K12C, was 9°, similar to other non-K12 sequences and for K12MC was 124.6°. For the tilt parameter, at the fifth base pair step, the median angle for all sequences except K12 was − 0.5°, for K12 was 57.7° and for the altered sequences K12C and K12MC was − 1.7° and 48.2 ^o^ respectively. As both control sequences (K12C and K12MethC clustered with all of the original controls we conclude that the distortion is caused by the ligand sequence complex and not the sequence alone.

## Discussion

We have previously carried out a comprehensive survey of the mutational patterns observed within the Ras genes and questioned the role DNA sequence context plays in determining within cancer differences between isoforms^7^. Why in smoking related lung tumours, G:C > T:A transversions predominate at *KRAS* codon 12 but are almost never observed at codon 12 in *HRAS* or *NRAS,* is unknown. Previously we studied the *TP53* gene and the effects of local sequence context on BPDE adduct-induced structural distortion at mutation sites in lung cancer, using molecular dynamics methods [47]. In this present study, we apply the same approach to explore the interplay between sequence context and differential structural distortion at codons 12, 13 and 14 across *RAS* isoforms.

The sequence context in DNA dictates local 3D structure and flexibility of the helix is dependent upon the sequence of bases in the helix [26]. Studies have attempted to elucidate the rules of this sequence dependence, and how sequences impact upon DNA replication, repair and mutation rates [27]. A number of studies have determined the effects local sequence has on damaged base conformation [28], influence of mutagenic potency [29, 30], as well as on the effects of bending DNA [31]. Despite many in silico and in vitro studies the exact relationships between DNA structural changes and mutation likelihood are still unclear.

Differences in DNA sequence between the Ras isoforms may influence the repair efficiency when the same carcinogen adduct occurs. The specificity and mechanisms of NER have been shown empirically and using modelling methods to be affected by base sequence context [32]. Furthermore, differing sequence can influence the rate of repair [33] and the degree of sequence dependent structural distortion [34]. Again, experimental and theoretical studies show the importance of distortions such as kinks, distortions in hydrogen-bonding and flipped nucleotides but the specific way in which these changes affect DNA structure and mutation/repair is unclear [27, 35–39]. The rate by which the NER repair mechanisms excise different bulky adduct lesions such as BPDE in different sequence contexts has been shown to vary greatly [1–4, 40]. In the NER pathway, the heterodimeric XPCRAD23B protein recognizes local distortion and destabilization of the DNA following lesion formation and base adduction. XPC-RAD23B binds to the damaged site causing both DNA strands to separate prior to replacement of nucleotides. Cai *et al*. have shown that the yeast recognition factor structure (*S. cerevisiae* NER recognition factor Rad4/Rad23, a homologue of XPC-RAD23B), following crystallization, points towards local thermodynamic stability at the site of the adduct and strongly influences whether the local region has a high or slow rate of repair [41]. The rate of repair is also influenced of course by the particular adduct. Tang and co-workers who found that repair of BPDE adducts at codon 12 of *KRAS* was slower and thus inefficient compared to *HRAS* and *NRAS *[19]. Furthermore, it was found that BPDE adducts at major mutation hotspot positions in the *TP53* gene are also regions of slow repair relative to other adduct sites [1]. A number of these BPDE adduct sites are associated with low DNA curvature which is sequence dependent[2]. Thus, local and more distal sequence context differences in Ras isoforms could result in differences in tertiary structure that significantly influence repair efficiencies. More distal neighbouring bases have also been shown to influence the rate of nucleotide excision repair at a site with a DNA lesion [42].

When data from NER assay results are combined with structural information derived from MD simulations, one can observe the variation in stacking stabilization between different types of adducts and different sequence contexts [43]. The effects of sequence context on BPDE adducts also correlate with the ability of NER [28, 44]. Therefore BPDE, as a well-characterized bulky adduct, is a model mutagen that can provide valuable mechanistic information when trying to assess variance and type of structural distortion due to sequence context in relation to repair rate at mutation hotspot sites having a wealth of available data [41, 45, 46].

Previously, we have used in silico molecular modelling techniques to study lung cancer mutation sites in the *TP53* gene and showed that the lung cancer specific hotspot at codon 157 was structurally distorted in a different way to other mutation hotspot and non-hotspot sequences when a BPDE adduct was present [47]. Along with Cai et al., we also showed that more distal bases must affect the type of DNA structural distortion when a bulky adduct is present as well as nearest base neighbours [13, 48]. In this current study, we explored how differing sequence contexts at mutation hotspots in the *RAS* isoforms affect the degree and types of local DNA structural distortion caused by adduct formation by BPDE at guanines.

The six eleven base pair sequences contain different three base central motif bar H12 and H13 which contain the same CGG triplet (both codons, however, have different neighbouring bases). The BPDE adduct on the guanine in each sequence studied is positioned in the minor groove and from here it interacts with a number of adjacent bases. By analysing seventeen helical parameters through multivariate statistics for each of the *RAS* hotspot and non-hotspot sites we were able to the describe changes in the structure of the DNA helices post BPDE adduct formation. MFA applied to the structural data then provided an indication of the overall degree of distortion induced by the BPDE adduct in each of the sequence contexts across the entirety of each sequence. MFA revealed that the buckle and tilt parameters greatly contribute to the different distortion seen between adducted K12 and other sequences including the strong BPDE binding site at K14 which had little distortion from a standard helical shape. Both the breaking and disturbing of hydrogen bonds has an impact on the buckling at a base pair. The impact of BPDE at the adduct site causes the base pair to move from pointing towards the centre of the helix, in a traditional Watson–Crick bonding pair, to point towards either end of the helical structure. The direction of distortion for the adducted K12 sequence was towards the 5’ at the fifth base. All other sequences showed very little distortion from their control (non-adducted) counterparts at the fifth base but an increase at the sixth base. This suggests the 5’ adjacent base (fifth base in sequence) in the K12 sequence, which is the only sequence studied to have a 5’ thymine, is having an impact on the helical distortion. Tilt describes a rotation about the short axis of the base pair. This change in tilt causes an immediate effect upon the DNA structure, and the shifting of the bases this way causes an atomistic clash with the base on the opposing strand, causing the DNA structure to change to eliminate this clash. The tilt parameter shows a lower angle change at the hotspot region but has a greater structural impact across the whole sequence. This destruction of helical structure is supported by the way in which the tilt parameter deviates and destroys the structure of surrounding base pairs. Due to these wider sequence changes, the tilt parameter could prove to play an important role in creating a statistical model for predicting overall structural damage on new sequences, as sequence context is clearly an important component in determining the level of changes at each base.

Hydrogen bond quality index (I_H_) is another interesting measurement of the differences between the K12 sequence and all other DNA sequences in this study. K12 is the only sequence where the hydrogen bond index deviates further from the ideal Watson–crick base pairing at the sixth, adducted, base. K14 showed the greatest decrease in I_H_ after adduction. This change from ideal Watson–Crick bonding was, however, not significant and may not relate to a great stabilisation in DNA structure. All other sequences showed only a small decrease in I_H_ with absolute changes of less than 0.04. K14 has the highest repair rate of all sequences [19] so this may be a significant parameter when considering DNA distortion. Interestingly, we have previously observed non-mutation hotspot sites in the *TP53* gene to have the greatest absolute change in I_H_ [47]. Therefore, we are cautious about drawing conclusions about the use of I_H_ when trying to correlate DNA structural distortion, repair and mutability.

K14 has been shown to have a BPDE guanine binding rate roughly equal to that of K12 but that adduct levels are reduced at these sites in *NRAS* and *HRAS *[19]. However, the repair rate of K12 is hypothesised to be the cause of the differing cancer mutation rate [19]. There are a number of structural differences that may contribute to this differing repair rate. BPDE is known to cause base substitutions which are most commonly G to T transversions and often observed at CpG methylated sites [49]. This may be due to the hydrophobicity of the methyl group in the methylated cytosine which may enhance the adduct formations at these sites. In K12 the adducted guanine is preceded by a thymine base. Thymine is a deaminated 5-methyl cytosine and therefore structurally similar. In both bases there is a methyl group at the C5 position. This methyl group sits in the minor groove where it interacts with the large bulky adduct (BPDE). Once the adduct has formed, adjacent sequence context then could influence the degree of structural distortion, reparability and ultimately the mutation rate. The presences of a methyl-group in thymine or methylated-cytosine has been shown to affect the fine structure of DNA [21–23], Liebl et al. employed molecular dynamics simulations to investigate the impacts of these sugar groups and showed the 5’ neighbouring sugar to be the main cause of any influence on backbone changes to the DNA sequence [50]. They also showed the methyl sugar group to cause a clash which results in an increase in local DNA flexibility.

**Fig. 5:**
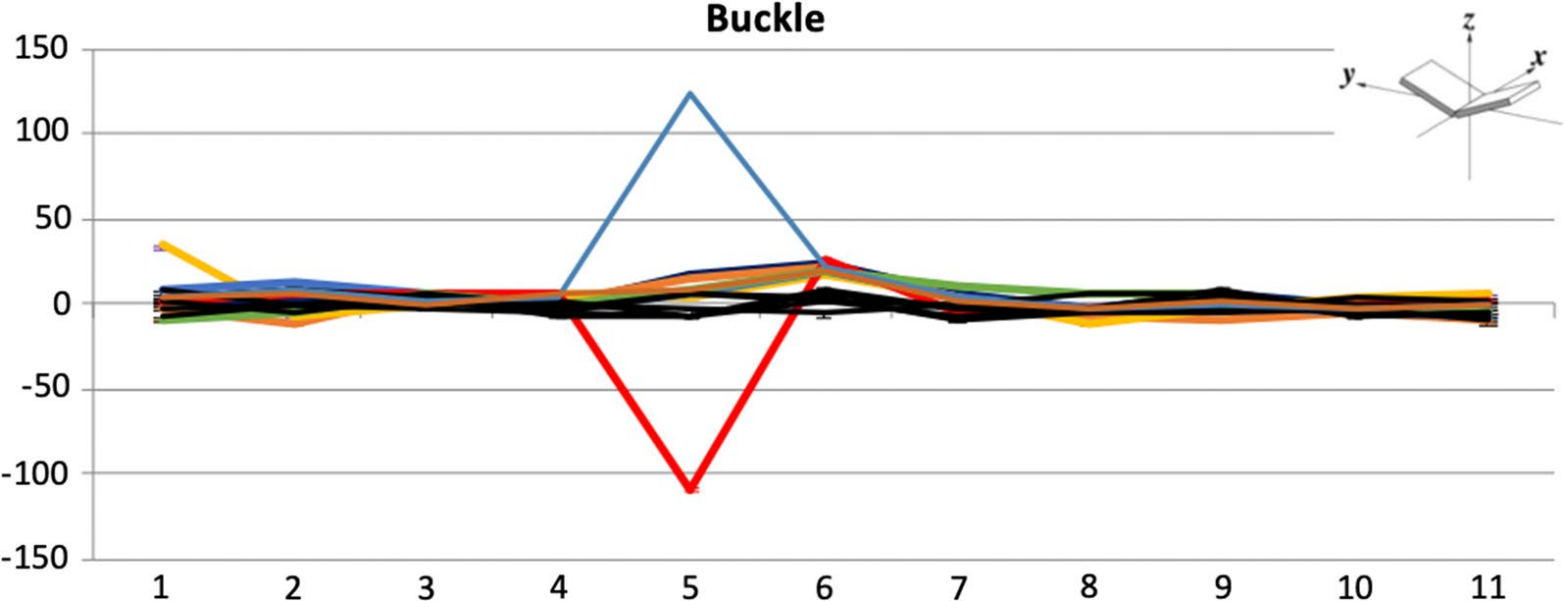
The average buckle at all points for all sequences including K12 in red, K12MethC in Blue and K12C in yellow. Parameter schematics re-printed, with permission, from [25] Control sequences are black, K14-orange, K13-purple, H12-indigo, H13-brown, K12-brown and N12-green

Combining what is known about the *RAS* gene mutation frequencies through the COSMIC database [14] and the DNA structural analysis carried out here, we hypothesize that although there is high binding of BPDE at both KRAS codons 12 and 14, G:C > T:A substitutions occur at a higher rate at codon K12 due to structural distortion difference. We explored whether the thymine base which precedes the adducted guanine in K12 causes more severe distortion in this sequence relative to others. We found that structural distortion of K12 changes when the 5’ thymine is altered in the sequence to a cytosine (K12C) prior to modelling. The fact that K12C groups with all other sequence studied provides evidence that the methyl group in the thymine contributes to the increased level of distortion at K12. Furthermore, when the 5’ thymine is replaced by a methylated cytosine the level of distortion is much greater (Fig. 5). Taking all of this into consideration, we hypothesise it is the thymine as the 5’ base to the adducted guanine in K12 which causes the increased distortion patterns in K12 compared to all other sequences studied. Specifically, it is the methyl group within the thymine base which interacts with the BPDE adduct as it sits in the minor groove on the DNA helix.

Data combined from this study and others investigating DNA repair in *RAS* genes suggests that increased adduct formation and relatively poor repair renders *KRAS* codon 12 more likely to end up mutated leading to a higher *KRAS* mutation rate observed in cancers. Although our data represents one mutagen, Ras isoform-specific differences in rates of DNA damage and repair have been identified for other carcinogens including N-acetoxy-2-acetylaminofluorene (NAAAF) that have different modes of binding to the target guanine.

## Conclusions

It is clear that the three RAS genes represent an excellent comparative model system for future investigation of the underlying genetic or epigenetic mechanisms leading to mutational spectra and hotspots. The availability of the entire genomes of cancer and non-cancer patients is rapidly increasing, and thus the wealth of disease specific mutation data. This provides the ability to perform comprehensive analysis of a great number of mutational hotspots, and the sequence context within which they occur. Further work should be done to understand the effect of individual base pair parameters across a host of sequences and adducts. Combine this with the increase in power and availability of high-performance computing, will allow methods like those discussed here to be used to help elucidate the reasons behind unique mutation hotspots in cancer types and thus increase our understanding of the aetiology of the disease. With wider scanning of DNA sequence contexts this could in the future lead to mutation screening programs of both possible new drugs or perhaps toxicological compounds. Further work should also be done to understand the effect of individual base pair parameters across a host of sequences and adducts.

## Methods

### DNA sequences

Six 11-mer duplex DNA sequences, encompassing either mutation hotspot or non-hotspot codons in lung tumours (Table 1) were created using Discovery Studio (https://www.3ds.com/products-services/biovia/products/molecular-modeling-simulation/biovia-discovery-studio/), this process involves switching the nucleobase only with the backbone of the DNA strand remaining fixed. Discovery Studio was also used to create the methylated cytosine in the same way. The middle base in each sequence is a guanine that is known to bind BPDE and form a bulky adduct in bronchial epithelial cells [19]. Simulations were performed on both adducted and non-adducted sequences to determine the degree of structural distortion after an adduct has formed. The COSMIC database [14] was searched to determine whether a mutated guanine within a sequence was a lung cancer mutation hotspot. G:C > T:A substitutions were counted for tumours designated in the database as non-small cell carcinoma, adenocarcinoma and squamous cell carcinoma and only recorded if occurring at a guanine that is a known BPDE binding site. The frequencies of G:C > T:A substitutions at each guanine are shown in Table 1. A guanine was designated as containing a lung cancer mutation hotspot if the observed number of G:C > T:A substitutions recorded at this site in the database was significantly increased relative to an expected number of mutations of the same type. In exon 2 the *KRAS, NRAS* and *HRAS* isoforms there are 32, 36 and 55 guanines in the coding strand. If we assume that mutations are equally likely in every guanine then the distribution of G:C > T:A substitutions in each isoform have probabilities of 0.031, 0.028 and 0.012 at each position respectively (*P*). A guanine is then classified as a hotspot if the number of mutations (*N*) at any given site is in excess of that predicted by a binomial distribution with a probability of *P*. The probability of finding *N* G:C > T:A substitutions at a site was calculated using an exact binomial test and compared to *P* with a Bonferroni correction.

**Table 1:**
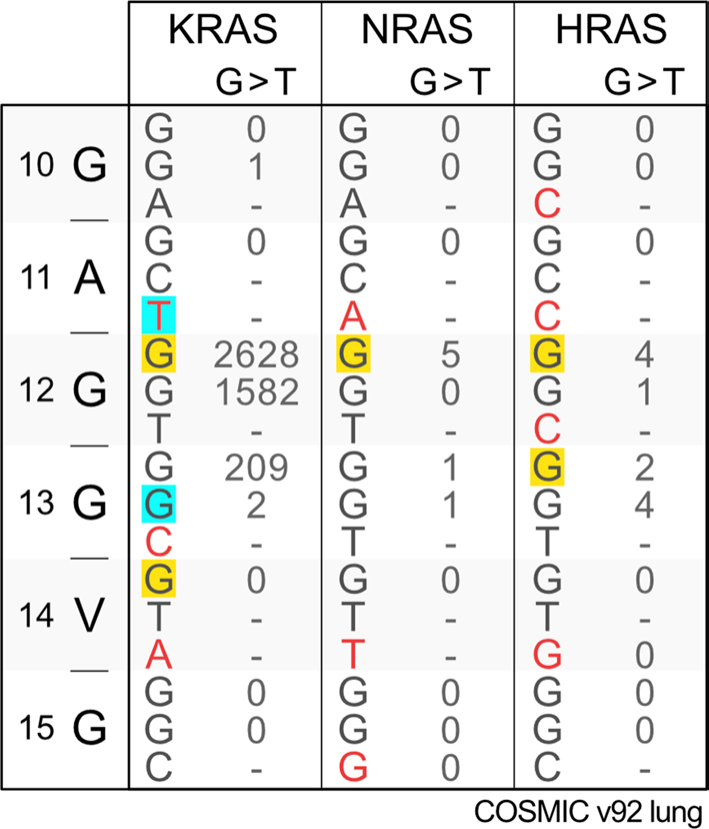
Ras isoform nucleotide alignments and mutation frequencies

Variant bases between isoforms are highlighted in red. Smoking-associated BPDE adducts result in G > T transversions, and analysis of lung cancer samples in the COSMIC genomic database reveals that these are prevalent only in the KRAS gene. Codon number and amino acid single letter code for the RAS isoforms can be seen in column one. To compare the influence of BPDE adducts on Ras DNA structure, 11 base sequences centred on the first guanine of codons 12, 13 and 14 were used in Molecular Dynamics simulations. Bases highlighted in yellow were compared ± BPDE adducts whilst those highlighted in blue were varied for other bases and/or methylated in the modelling

### Molecular dynamics (MD) simulations

All simulations were carried out, in triplicate, using the GROMACS package [51] using the Amber99 force field [52] with modifications [53, 54]. Forcefield parameters for BPDE bound to guanine, and methylated cytosine were created as detailed in [47]. The simulations were all carried out in the NPT ensemble, with periodic boundary conditions, at a temperature of 300°K, and a pressure of 1 atm. The DNA structures were placed in a cubic box, solvated using TIP3P water, neutralised with the appropriate number of Na + ions prior to simulation. For all simulations, v-rescale temperature coupling thermostat was applied and Particle-mesh Ewald (PME) was applied to long range electrostatics. Each simulation was performed using a three-step process: steepest descent energy minimisation with a tolerance of 1000 kJ^−1^ nm^−1^ and a cut off of 3000 steps. Next a pre MD run (PR) with 25,000 steps at 0.002 fs time step per second per step making a total of 2500 ps; and an MD stage run for a total of 100 ns.

### Hydrogen Bond Quality Index

The Hydrogen Bond Quality Index (*I*_*H*_) was used to determine, for a selected base pair, deviations of hydrogen bonds from ideal Watson–Crick hydrogen bonding [24]. The equation to derive *I*_*H*_ is as follows:

*I*_*H*_ = ∑*D* − *H*…*A* [24].

In an ideal C:G bond, distances would be: O6 (G) to N4 (C) = 2.91 Å; N1 (G) to N3 (C) = 2.95 Å; and N2 (G) to O2 (C) = 2.86 Å. The ideal bond angle is always 180°. A perfect Watson–Crick bonding pair would result in the I_H_ equation equalling 0. Median I_H_ values across simulation time points were calculated at the adducted base pair for each sequence.

### DNA structural parameters measured

Variations in seventeen DNA structural parameters were measured. These parameters are made up of six intra-base pair parameters—shear, buckle, stretch, propeller, stagger and opening and six inter-base pair parameters—shift, tilt, slide, roll, rise and twist. Finally base pair axis parameters X and Y displacement, as well as axis-bend, inclination and tip, were also measured and describe the geometry of the base pair relative to the helical axis. Parameters were measured using open source Curves + and Canal software [55]. Helical parameter data for each base pair were compared to published reference values [56].

### Statistical analysis of data distributions

Each data distribution was tested for normality using the Anderson–Darling test. All structural parameter data distributions were observed to be non-normal and a Mann–Whitney *U* test used to determine any statistical differences between adducted and control sequence data.

### Multivariate statistical analysis

Multivariate statistical analysis (MVA) was applied to the output structural data to interpret the distortion caused by adducts, as previously described [47] relative to the same non-adducted sequence as well as between sequences. Multiple factor analysis (MFA) is an extension of principal components analysis (PCA) designed to analyse multiple data tables that measure sets of variables collected on the same observations. In this study, the observations are the different DNA sequences and a variable set represents the measurements across the sequence for a given structural parameter. Thus, in total, there were seventeen variable sets representing each structural parameter. A variable set contained either 10 measurements for intra-base pair parameters or 11 measurements for each of the other parameters. MFA elucidates the common structures present in all or some of these sets and the method performed in two steps. MFA was performed using the ‘mfa’ function from the FactorMineR [57] package in R statistical environment.

## Supplementary Information

**Additional file 1** Excel sheet containing the average and standard deviations for each base from the curves+analysis, individual sequences can be found in each excel tab and different parameters found in each row

**Additional file 2: Table S1**. RAS DNA sequences used for molecular dynamics simulations (adducted guanines in yellow). **Figure 1. **schematic of the Benzo-a-pyrene Diol Epoxide structure.** Figure 2**. Most common structures from each simulation, found through the gromacs cluster command. A-K13mS B-H12S C-H13S D-N12S.** Figure S3**. RMSF per residue for each of the sequences. **Figure S4**. Zoomed in K12 BPDE T5 positioning

## Data Availability

The datasets generated and/or analysed during the current study are available in the COSMIC repository, https://cancer.sanger.ac.uk/cosmic.

## Abbreviations

BPDE: Benzo[a]pyrene diol epoxide
NER: Nucleic Excision Repair
BaP: Benzo[a]pyrene
10S: (K)-7S,8R,9R,10S + anti-B(a)PDE enantiomer
MD: Molecular dynamics
RMSD: Root mean square deviation
MFA: Multiple factor analysis
I_H_: Hydrogen bond quality index
K14: *KRAS* Codon 14
K12: *KRAS* Codon 12
K12MethC: K12C sequence
NAAAF: N-acetoxy-2-acetylaminofluorene
MVA: Multivariate statistical analysis
PCA: Principal components analysis
