# Supplementary material for: Carcinogen-induced DNA structural distortion differences in the RAS gene isoforms; the importance of local sequence
Source: BMC Chem. 2021 Sep 14;15(1):51. doi: 10.1186/s13065-021-00777-8 (PMC8439098; doi:10.1186/s13065-021-00777-8)
Supplement: Supplementary file 2 — Additional file 2: Table S1. RAS DNA sequences used for molecular dynamics simulations (adducted guanines in yellow). Figure 1. schematic of the Benzo-a-pyrene Diol Epoxide structure. Figure 2. Most common structures from each simulation, found through the gromacs cluster command. A-K13mS B-H12S C-H13S D-N12S. Figure S3. RMSF per residue for each of the sequences. Figure S4. Zoomed in K12 BPDE T5 positioning [file 13065_2021_777_MOESM2_ESM.docx]

**Supplementary information**

Supplementary data 1 is an excel file which contains all averages and standard deviations for all curves+ data for each of the sequences (control and adducted).

Individual worksheets are present for each sequence and named as such. Parameters can be found in column B going down and each base pair of base pair step’s average and standard deviation is found from left to right.

| Codon | Sequence ID | *P-* Value* | RMSD** | |
| --- | --- | --- | --- | --- |
|  |  |  | Control | Adducted |
| ***KRAS*** Codon 12 | K12 | <0.001* | 0.181 (0.02) | 0.293 (0.05) |
| ***KRAS*** Codon 13 | K13m | NS | 0.186 (0.02) | 0.234 (0.02) |
| ***KRAS*** Codon 14 | K14 | NS | 0.187 (0.02) | 0.230 (0.02) |
| ***NRAS*** Codon 12 | N12 | NS | 0.185 (0.02) | 0.220 (0.01) |
| ***HRAS*** Codon 12 | H12 | NS | 0.178 (0.02) | 0.239 (0.02) |
| ***HRAS*** Codon 13 | H13 | NS | 0.186 (0.02) | 0.240 (0.02) |
| ***KRAS*** *Codon 12*  *C at 5^th^ base* | K12C | - | - | - |
| ***KRAS*** *Codon 12*  *Methylated C at 5^th^ base* | K12CM | - | - | - |

Supplmentary Table 1 *RAS* DNA sequences used for molecular dynamics simulations (adducted guanines in yellow).

*P* indicates the *P-Value* that determined whether the adducted guanine site is a significant G:C>T:A mutation hotspot in lung cancer. NS = not significant at the 5% significance level. N/A = not available. ** Standard deviations for RMSD values are in parentheses


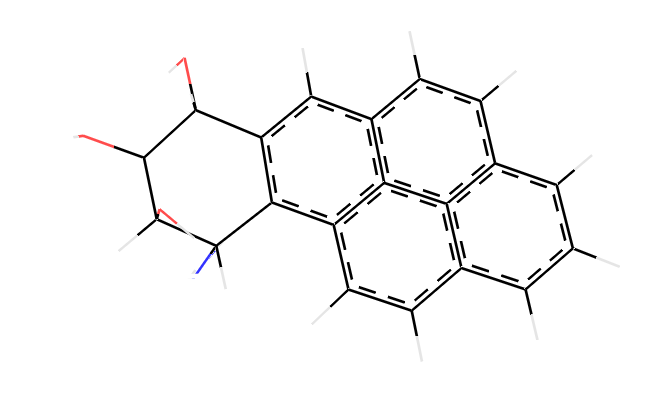


Supplementary Figure 1 schematic of the Benzo-a-pyrene Diol Epoxide structure.


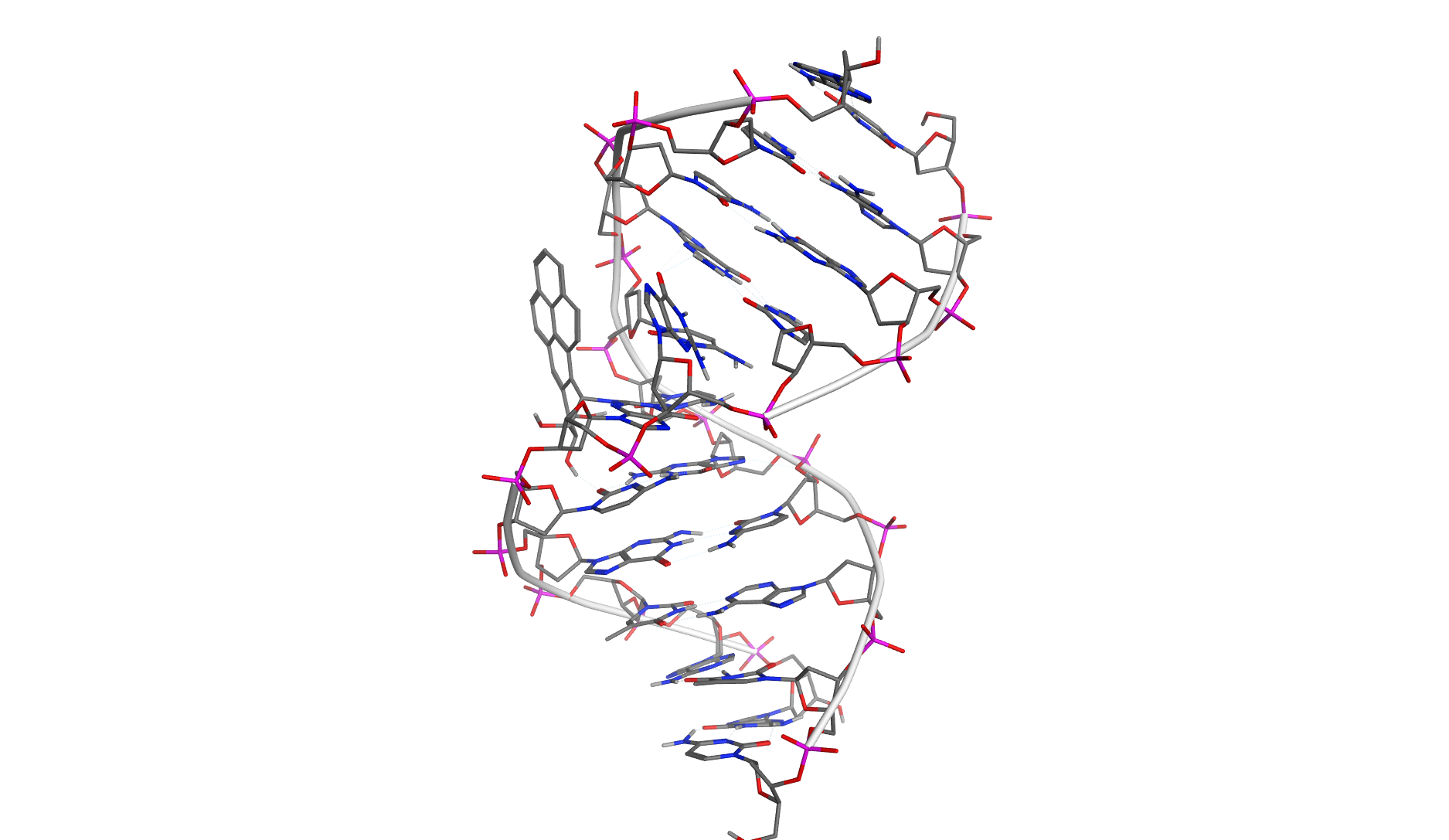

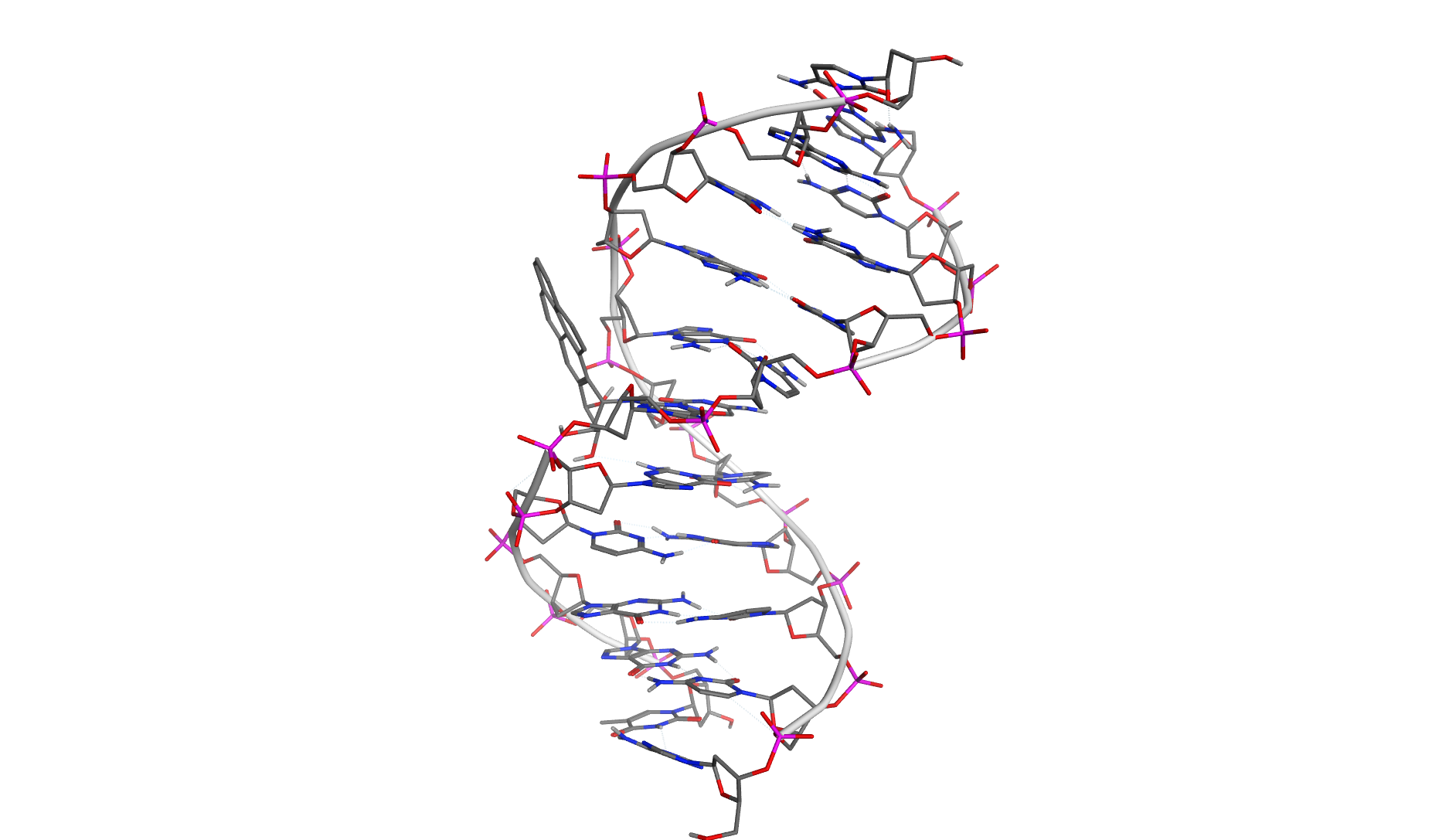


B

A


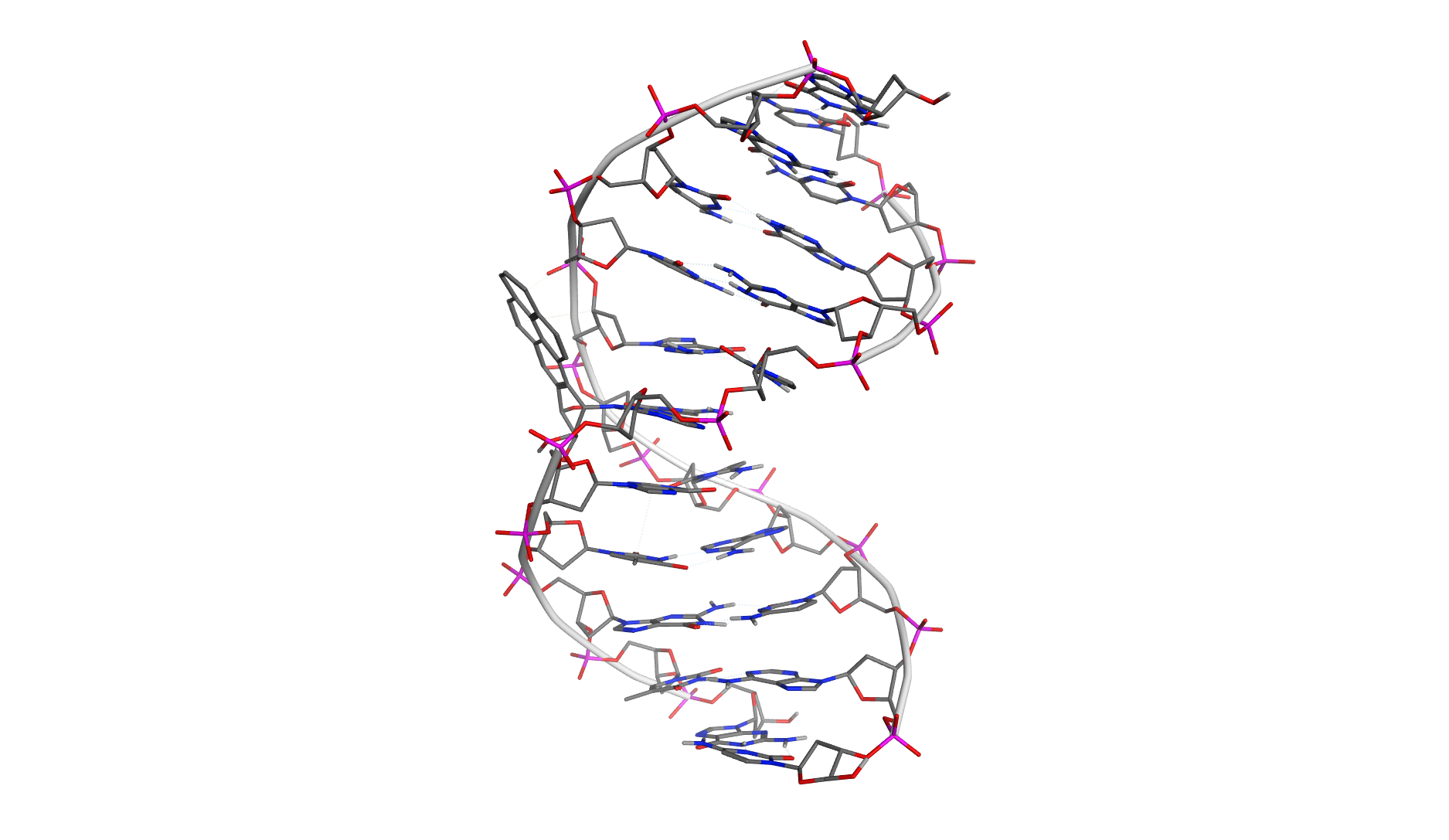

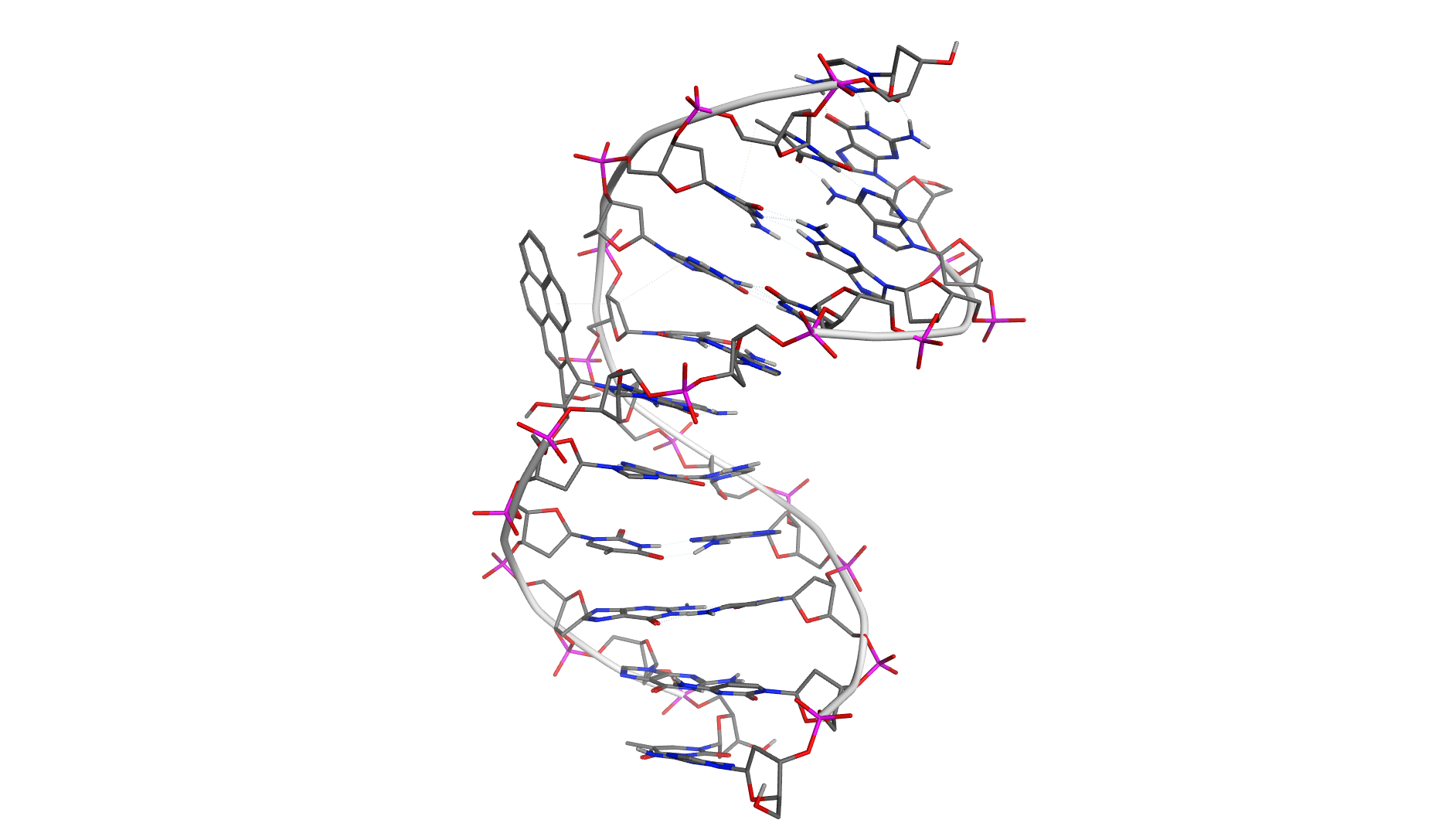


D

C

Supplementary Figure 2 - Most common structures from each simulation, found through the gromacs cluster command. A-K13mS B-H12S C-H13S D-N12S

Supplementary Figure 3 - RMSF per residue for each of the sequences


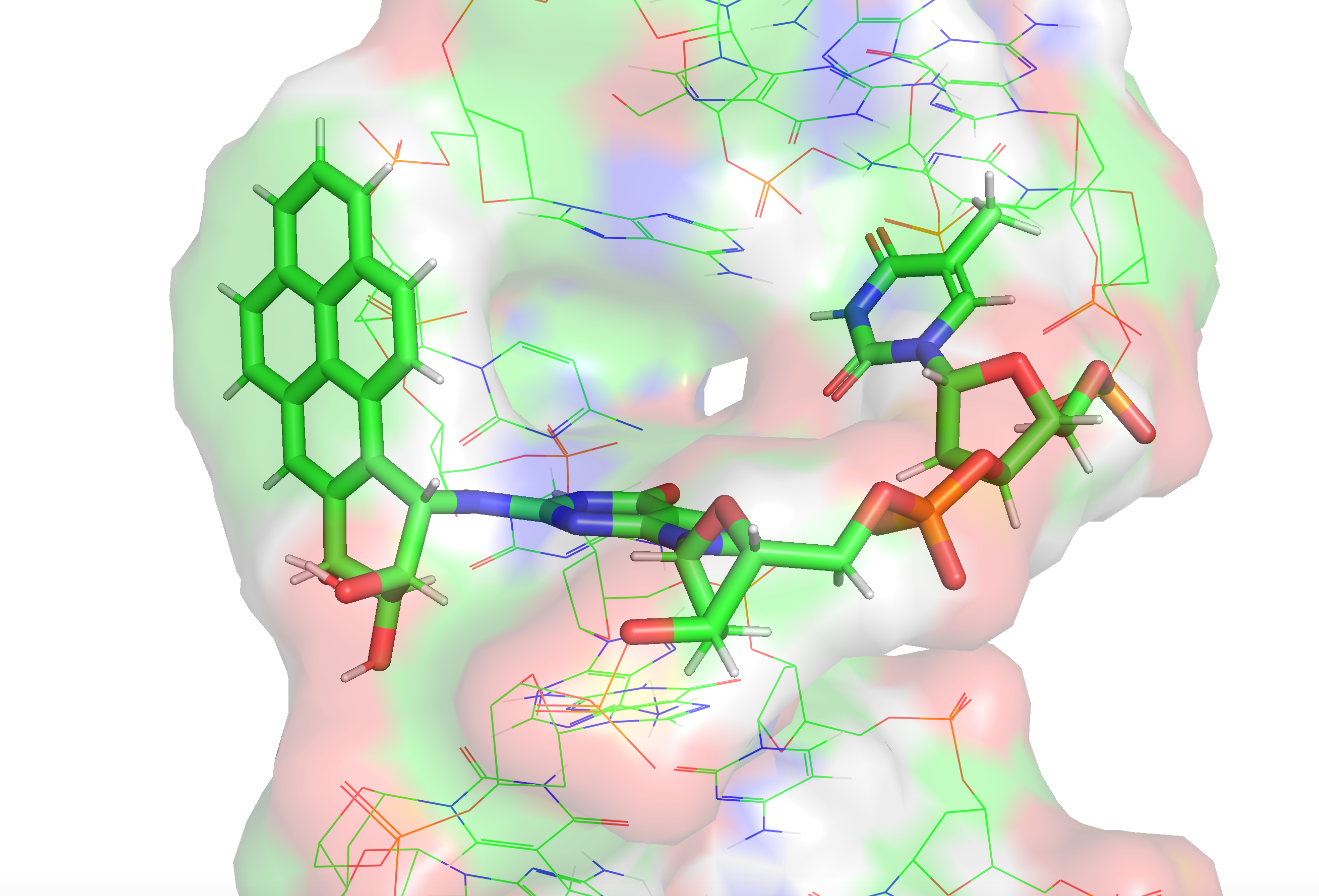


Supplementary Figure 4 - Zoomed in K12 BPDE T5 positioning.
